# Protective effects of triptolide against oxidative stress in retinal pigment epithelium cells via the PI3K/AKT/Nrf2 pathway: a network pharmacological method and experimental validation

**DOI:** 10.18632/aging.205570

**Published:** 2024-02-21

**Authors:** Fuying Pan, Qinxin Shu, Hao Xie, Long Zhao, Ping Wu, Yong Du, Jing Lu, Yuxia He, Xing Wang, Hui Peng

**Affiliations:** 1 Department of Ophthalmology, The First Affiliated Hospital of Chongqing Medical University, Chongqing 400016, China; 2 Chongqing Key Laboratory of Ophthalmology, Chongqing Eye Institute, Chongqing 400016, China

**Keywords:** triptolide, oxidative stress, PI3K/Akt/Nrf2 pathway, network pharmacological analysis

## Abstract

Purpose: Among aging adults, age-related macular degeneration (AMD), is a prevalent cause of blindness. Nevertheless, its progression may be halted by antioxidation in retinal pigment epithelium (RPE). The primary effective constituent of Tripterygium wilfordii Hook. F., triptolide (TP), has demonstrated anti-inflammatory, antiproliferative, and antioxidant properties. The mechanics of the protective effect of triptolide against the oxidative damage in retinal pigment epithelial (RPE) were assessed in this study.

Methods: ARPE-19 cells were pretreated with TP, and then exposed to sodium iodate (SI). First, cell viability was assessed using CCK-8. Subsequently, we measured indicators for cell oxidation including reactive oxygen species (ROS), catalase (CAT), superoxide dismutase (SOD), and malondialdehyde (MDA). Then, we used network pharmacological analysis and molecular docking to explore the signaling pathway of TP. Last, we used western blot, ELISA, and immunofluorescence assays to clarify the potential mechanistic pathways.

Results: The network pharmacology data suggested that TP may inhibit AMD by regulating the PI3K/Akt signaling pathway. Experimental results showed that the potential mechanism is that it regulates the PI3K/Akt pathway and promotes Nrf2 phosphorylation and activation, thereby raising the level of antioxidant factors (HO-1, NQO1) and reducing the generation of ROS, which inhibit oxidative damage.

Conclusion: Our findings suggested that the effect of TP on SI-exposed RPE cells principally relies on the regulation of oxidative stress through the PI3K/Akt/Nrf2 signaling pathway.

## INTRODUCTION

Age-related macular degeneration (AMD) is a degenerative, chronic eye disease that occurs with aging and ultimately leads to severe sight loss. RPE are monolayer pigment cells that lie between the photoreceptors and Bruch’s membrane. It plays an essential functional role in keeping the retina stable and the photoreceptors functional [1]. Although the pathology of AMD has not been well comprehended, multiple factors have been suggested. Of these factors, RPE oxidation is a serious one [2, 3]. The accumulated and long-term oxidative damage to the RPE may lead to the progression of AMD. Thus, antioxidant therapy targeting RPE cells can be a potential method for treating AMD.

Oxidative stress has been thought to be one of the main contributors in the progression of AMD. An imbalance in the generation and clearance of oxygen-derived free radicals as a result of decreased activity of endogenous antioxidant enzymes or increased accumulation of reactive oxygen species (ROS) can result in oxidative stress [4, 5]. Studies have revealed that oxidative stress expedites the progression of neurodegenerative disorders that cause cognitive impairment, among other symptoms [6]. Oxidative factors, such as sunlight and aging, cause oxidative damage to RPE cells, generating drusen deposits between Bruch’s membrane and RPE. Recent research has emphasized that Nrf2 regulates inflammation and oxidative stress response by modulating the expression of genes encoding for anti-inflammatory and antioxidant factors, which additionally play an active role in some forms of oxidative stress and inflammatory diseases, such as AMD [7].

Several pieces of evidence have shown that the expression and activity of Nrf2 in cells decrease with aging, which means that Nrf2 is a vital regulatory factor in cell aging [8–10]. Additionally, research shows that by promoting heme degradation, the Nrf2-HO1 axis defends TJ integrity and RPE barrier function [11]. The upward increase of Nrf2 can maintain the retinal function by protecting the retinal cells from oxidative stress [12, 13]. Hence, Nrf2 enhancement is a key antioxidant approach for the treatment of AMD. The PI3K/Akt signaling pathway is associated with neurodegenerative diseases [14] and is also an important survival signaling pathway that regulates cellular defense against oxidative damage [15]. It has been studied that PI3K/Akt is a signaling molecule upstream of Nrf2 [16], and activation of PI3K/Akt reduces oxidative damage in cells and activates Nrf2, which upregulates HO-1 expression to protect cells [17–19]. Studies have shown that activation of PI3K/Akt by chlorogenic acid is protective against hydrogen peroxide-induced oxidative damage in MC3T3-E1 cells [20].

Lei Gong Teng is another name for the traditional Chinese herbal remedy Tripterygium wilfordii Hook F. (TWHF). Triptolide (TP) is a pharmacologically active component of this plant. Triptolide (TP) has various biological activities, such as antioxidant, anti-inflammatory, and antiproliferative activities, as well as immune regulation, which has attracted great attention [21, 22]. Research has shown that TP is a potential neuroprotectant that can improve neurodegenerative disorders by reducing the oxidative stress and the production of inflammatory cytokines. In the transgenic mouse model with ischemia-reperfusion (I/R) injury, the anti-inflammatory and antioxidant effects of triptolide have been demonstrated [23, 24]. In DHCA rats, TP was also reported to improve oxidative stress, neuroinflammation, and neurobehavioral functions [25]. However, relatively few studies have been conducted on using TP in AMD treatment, and the specific mechanism of TP is still not clear. Further investigation is needed to determine the exact protective role of TP against sodium iodate-induced oxidative stress in RPE, which effectively mimics several features of age-related macular degeneration [26]. This may provide a potential therapeutic strategy to control AMD.

In this study, we constructed a model for sodium iodate-mediated oxidative stress in RPE to investigate the protective effect of triptolide and its molecular mechanics. Through network pharmacology analysis, the potential mechanism and therapeutic target of triptolide in AMD were further investigated. These findings offer a rational theoretical basis for the use of TP and may uncover novel therapeutic targets and effective treatments for AMD.

## RESULTS

### Role of TP in cell viability and its antioxidative stress properties

**Figure 1:**
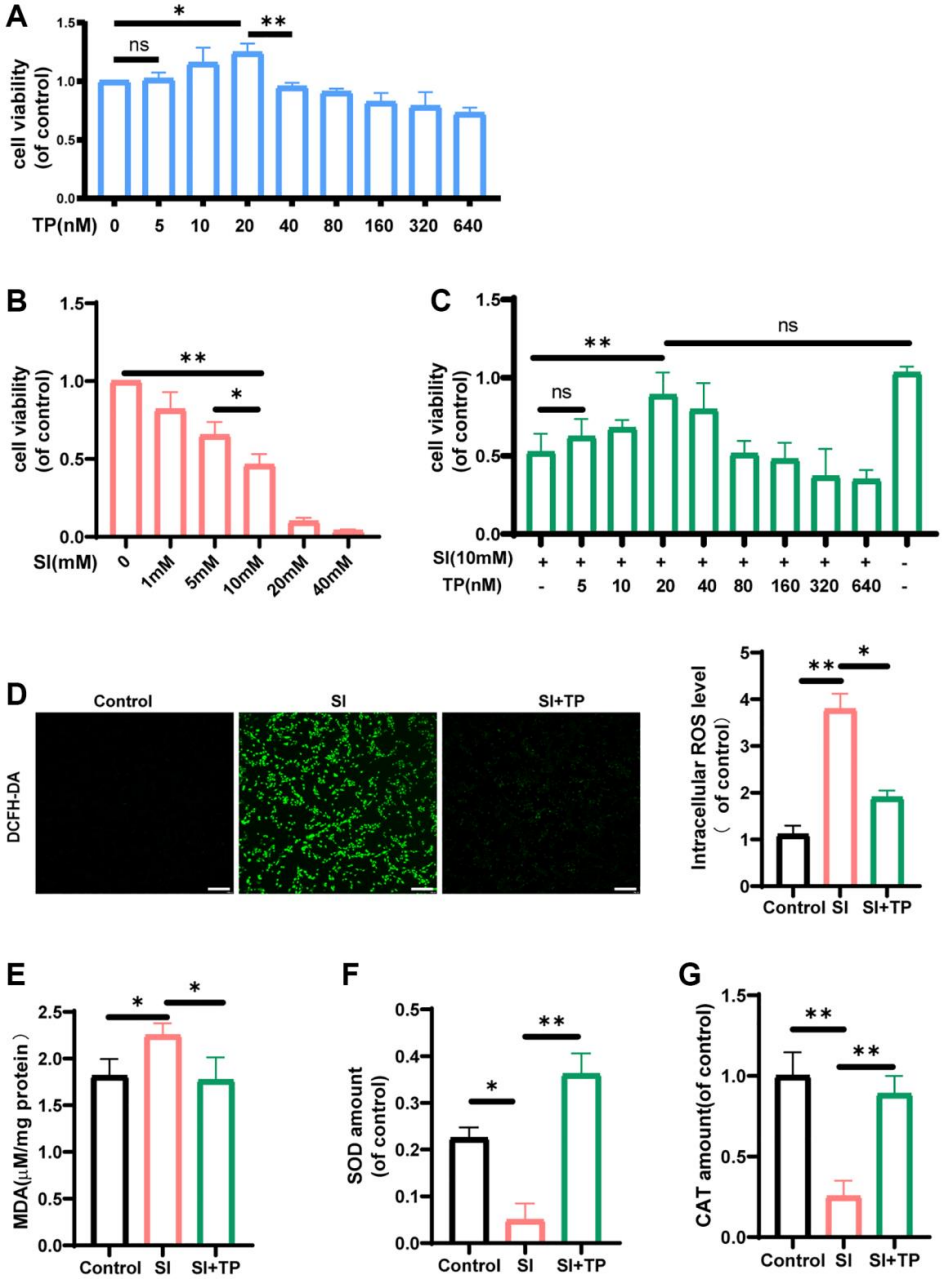
**The effects of triptolide on the levels of pro- and anti-oxidant factors and the cell viability.** The ARPE-19 cells were incubated with different concentrations of triptolide (TP) (**A**) or sodium iodate (SI) (**B**) for 24 h. (**C**) The ARPE-19 cells were treated with TP for different concentrations and then exposed to 10 mM SI for 24 h, *n* = 4. TP prevented the decrease in retinal pigment epithelial cell viability induced by SI. The fluorescence images of ROS were measured by a fluorescence microplate, *n* = 3. (**D**) The data showed that TP reduced the generation of ROS in ARPE-19 cells significantly. The amounts of MDA (**E**), SOD (**F**), CAT (**G**) in cell lysates were detected by a microplate reader using commercial kits. Data are shown as mean ± standard deviation (SD), *n* = 3; Scale bar, 300 μm. In all cases, the control is untreated retinal pigment epithelial (RPE) cells. Abbreviation: NS: not significant. ^*^P < 0.05. ^**^P < 0.01.

The sodium iodate-mediated oxidative stress in RPE can mimic many features of AMD. Thus, SI was used to simulate cellular oxidative damage. CCK-8 tests were used to assess the viability of cells. Before investigating the efficacy of triptolide, we determined its safety in ARPE-19 cells. The results of the CCK-8 tests (Figure 1A) suggested that concentrations of triptolide below 40 nM were safe for ARPE-19 cells. It has been discovered that in HepG2 cells, cell viability markedly decreases when the concentration of TP exceeds 40 nM [27]. To assess its protective effect, we pretreated ARPE-19 cells with TP at different concentrations. Then, ARPE-19 cells were cultured using different concentrations of SI for 24 hours. Figure 1B shows a dose-dependent decrease in cell viability. Some studies suggest that an acute nonlethal dose for extending treatment analysis may be selected at concentrations of 10 mM or lower of sodium iodate [28]. Based on the findings of the CCK-8 test, we selected a concentration of 10 mM (*p* < 0.01) to be administered in the sodium iodate group (SI group). Figure 1C shows 20 nM (*p* < 0.01) as the optimal concentration for TP treatment. Additionally, TP improved the cellular activity in SI-exposed ARPE-19 cells.

To better determine the protective effect of TP against the SI-mediated oxidative damage, we evaluated a series of indicators of oxidation and antioxidation. In contrast to the control group, as shown in Figure 1D, ROS increased (*p* < 0.01) in the SI group. However, in the TP-pretreated group (SI+TP group), the levels of ROS in ARPE-19 cells significantly decreased (*p* < 0.05). The results of the flow cytometric analysis were the same (Supplementary Figure 1A). Furthermore, in comparison with the control group, the MDA contents notably increased (*p* < 0.05) after exposure to SI (Figure 1E). Compared with the SI group, the TP-pretreated group showed significantly decreased levels of well-known antioxidative enzymes (*p* < 0.01), SOD (Figure 1F) and CAT (Figure 1G).

### Protective effects of TP on retinal pigment epithelial barrier function, mitochondrial membrane potential, and proliferation

**Figure 2:**
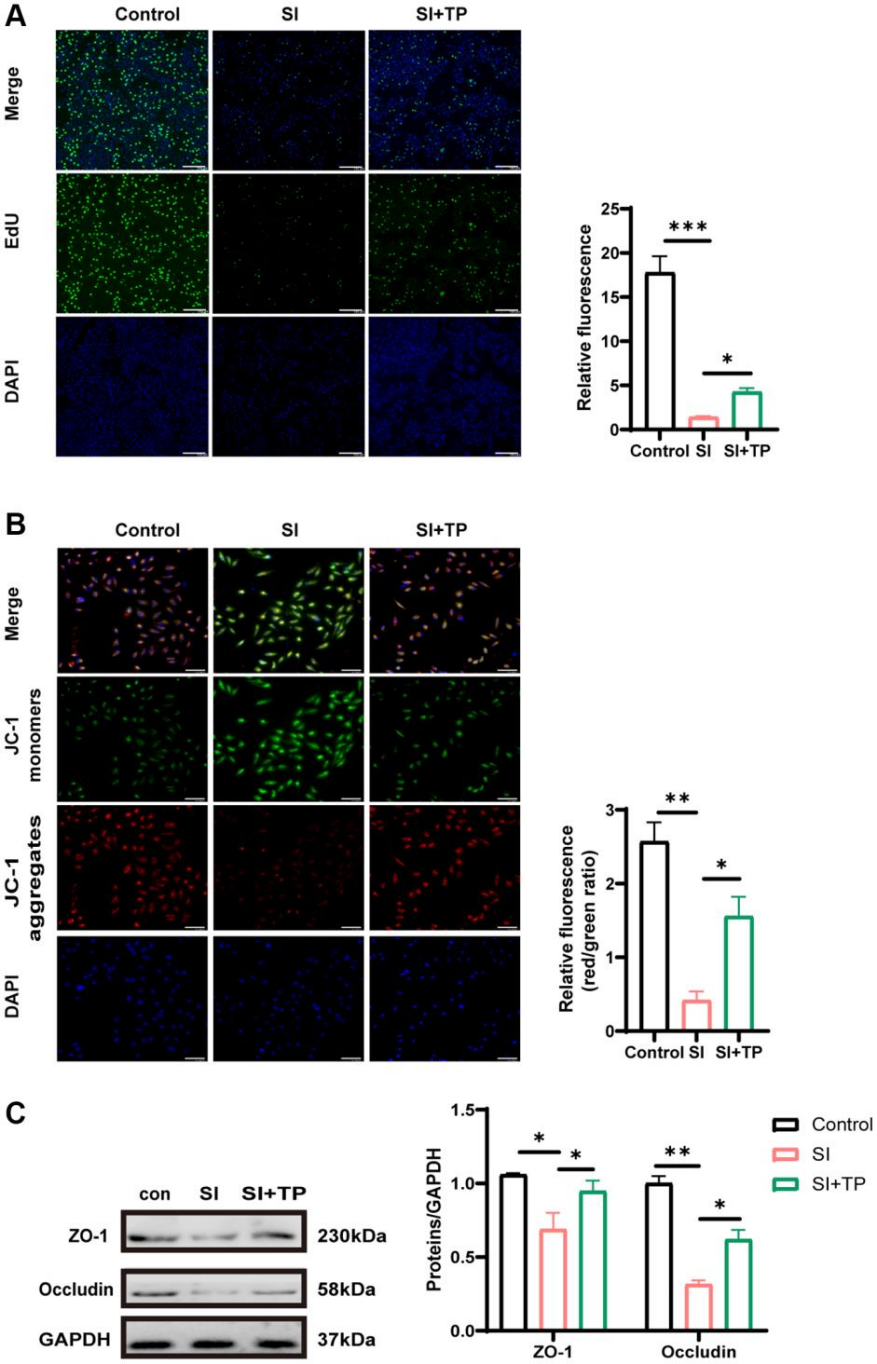
**Effects of TP on proliferation, mitochondrial membrane potential and tight junction protein.** (**A**) The EdU assay was used to analyze the proliferation-suppressing effect of SI on RPE cells. DAPI for nuclear staining (blue). EdU-positive cells (green) were counted to calculate the percentage. Scale bar, 200 μm. (**B**) Mitochondrial membrane potential was detected by the JC-1 fluorescence ratio. The transition from red fluorescence to green fluorescence represents the decrease of cell membrane potential. Scale bar, 200 μm. (**C**) Protein levels of occludin and ZO-1 were detected by Western blot with GAPDH as the loading control. The bar graphs show the results of analysis. Values are the mean ± SD; *n* = 3; ^*^P < 0.05, ^**^P < 0.01, ^***^P < 0.001.

To determine whether TP regulates ARPE-19 function, we examined the proliferation and mitochondrial membrane potential of ARPE-19 treated with or without TP *in vitro*. We found that co-treatment with TP and SI attenuated the antiproliferative effects of SI (*p* < 0.05) (Figure 2A). JC-1 fluorescence ratio was used to detect mitochondrial membrane potential. Mitochondrial depolarization is frequently quantified by comparing the red-to-green fluorescence ratio. The results of JC-1 staining showed that SI induced a dissipation of the mitochondrial membrane potential (*p* < 0.01), while TP ameliorated the mitochondrial depolarization (*p* < 0.05) (Figure 2B). These all demonstrated the protective potential of TP.

The tight junctions of the paracellular barrier serve as the cornerstone of any epithelial barrier. Oxidative damage to RPE frequently results in the disruption of the epithelial barrier function. Western blotting revealed the expression of two essential elements of the epithelial cytoskeleton, namely occludin and ZO-1. As depicted in Figure 2C, the expression of ZO-1 (*p* < 0.05) and occludin (*p* < 0.01) was notably decreased in the SI-exposed group, demonstrating that epithelial barrier function was significantly compromised after SI exposure. However, their expression in the TP-pretreated group (SI+TP group) was significantly elevated relative to the SI-induced group (*p* < 0.05). These results showed that TP exerts some protective effect on RPE tight junctions.

### Network pharmacology analysis and molecular docking

**Figure 3:**
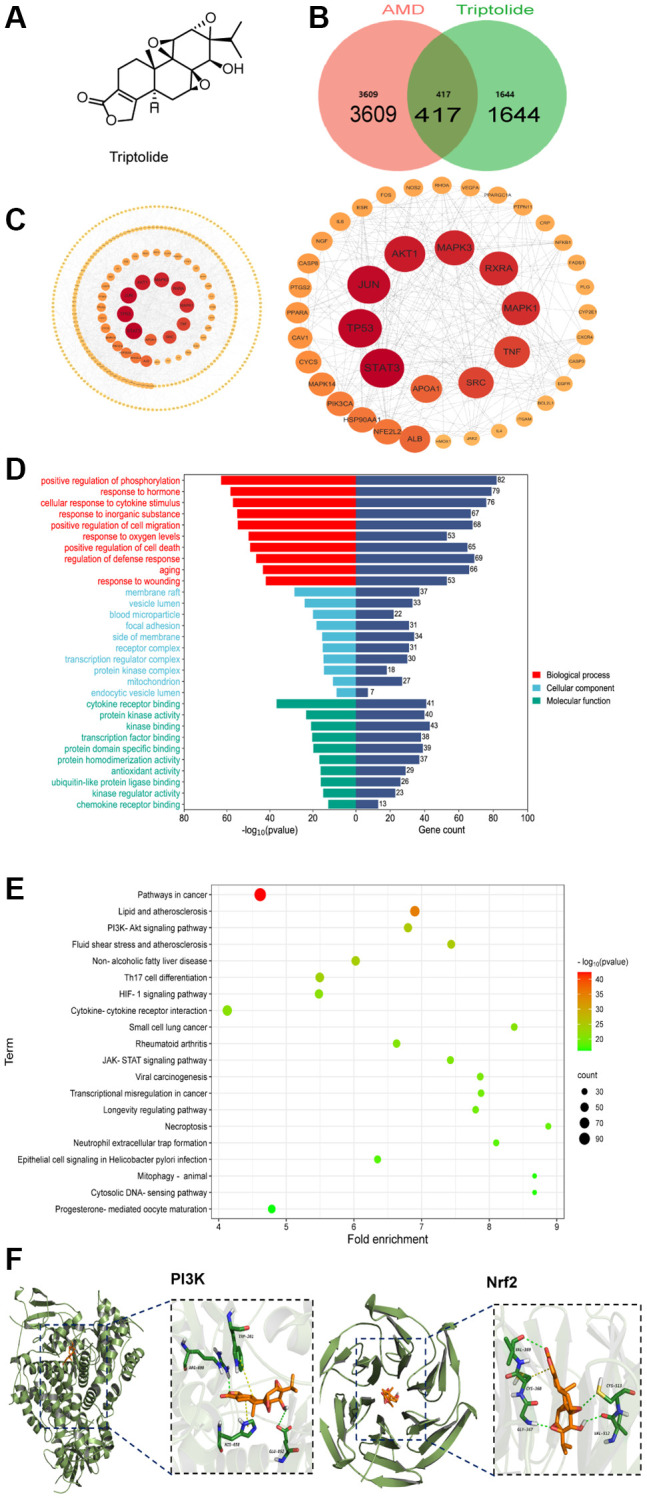
**The underlying target of triptolide by network pharmacology analysis.** (**A**) The chemical structure of TP. (**B**) The 417 intersection targets of TP against AMD (Venn diagram). (**C**) The crucial protein interaction network diagram. The larger the degree of the node in the graph, the darker the color and the larger the diameter of the node. (**D**) GO functional annotation for potential targets of TP on AMD. (**E**) KEGG enrichment analysis for potential targets of TP on AMD. (**F**) The molecular docking model of TP with PI3K and Nrf2.

TP is a natural compound with low molecular weight (360.4Da). Figure 3A shows its chemical structure. To delineate the protective mechanism of TP against AMD, we used network pharmacology analysis to predict its potential targets. Using an online Venn diagram tool, we identified 2061 TP-related targets, 2,026 AMD-related targets, and 417 cross-targets (Figure 3B). To establish the protein-protein interaction (PPI) network, the 417 genes were integrated into the STRING database. Using Cytoscape (v3.10.0) software, a PPI network, comprising of 409 nodes and 1950 edges, was reconstructed from the STRING database (Figure 3C). Using overlapping genes to enrich GO and KEGG, we could evaluate AMD-related signal transduction pathways and biological processes which may be influenced by TP. The result of GO enrichment includes three parts: molecular function, cell composition, and biological process. Figure 3D shows the bar graphs of the top 10 results from the three parts of GO enrichment. In Figure 3D, according to GO analysis, the target genes were mostly enriched in “positive regulation of phosphorylation” (biological process), “membrane raft” (cellular component), and “cytokine receptor binding” (molecular function). According to KEGG enrichment analysis, the target genes were shown to be implicated in a number of different pathways, including Th17 cell differentiation, the PI3K/Akt signaling pathway, HIF-1 signaling pathway, and longevity-regulating pathway. The PI3K-Akt signaling pathway showed much higher enrichment (Figure 3E). These results indicated that the PI3K-Akt pathway may be linked to the potential protective mechanism of TP against AMD. Nuclear factor erythroid 2-related factor 2 (Nrf2), as a transcription factor, regulates the expression of antioxidants, phase 2 detoxification enzymes, and other related proteins. Nrf2 is essential for the regulation of oxidative status. Thus, we focused on two hub proteins: PI3K and Nrf2. To anticipate the potential binding of TP to PI3K (PDB code: 1E7U) and Nrf2 (PDB code: 5CGJ), a molecular docking simulation was performed using AutoDock Vina. Triptolide binds to Nrf2 protein mainly through hydrogen bonding and conjugation. VAL512, CYS513, GLY367, and VAL369 can each form a hydrogen bond with triptolide, whereas CYS368 can conjugate with its cyclic structure. Additionally, RG690 and GLU852 can each form a hydrogen bond with triptolide, while TRP201 and HIS658 can form a conjugate with triptolide (Figure 3F). The calculated free energy of TP binding to Nrf2 is −9.9 kcal/mol, while that of its binding to PI3K is −9.0 kcal/mol, implying that TP has relatively good binding ability to these two molecules.

### Role of the PI3K/Akt pathway in TP-mediated cytoprotection against oxidative stress

**Figure 4:**
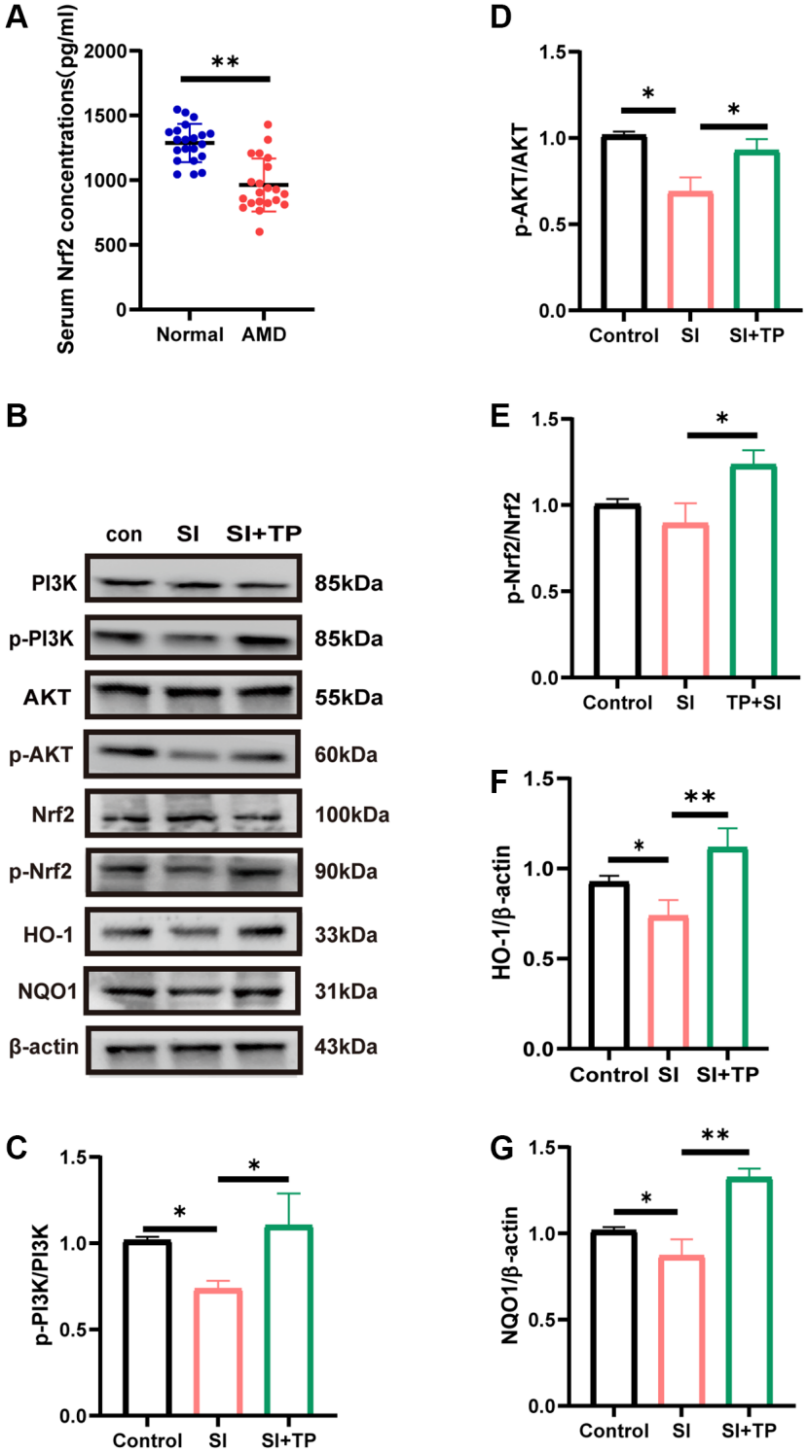
**Activation of the PI3K/Akt pathway was involved in the protective effect of triptolide on ARPE-19 cells.** (**A**) Serum Nrf2 levels in normal subjects and AMD patients detected by ELISA, *n* = 3. (**B**) Cells were treated with 20 nM triptolide (TP) for 6 h and then treated with sodium iodate (SI) for 24 h. Western blot analysis was performed using the corresponding antibodies. Quantitative analyses of p-PI3K/PI3K (**C**), p-Akt/Akt (**D**), p-Nrf2/Nrf2 (**E**), HO-1 (**F**) and NQO1 (**G**) by ImageJ software. Data are shown as mean ± standard deviation (SD) (*n* = 3); Abbreviation: NS: not significant. ^*^p < 0.05. ^**^P < 0.01.

The PI3K/Akt signaling pathway has been proposed to be essential for the regulation of the antioxidant function in RPE cells [29]. ELISA revealed that the level of Nrf2 in the serum of patients with AMD was significantly lower (*p* < 0.01) compared with controls (Figure 4A). Western blot was used to measure the protein levels for p-PI3K, PI3K, p-Akt, Akt, p-Nrf2, and Nrf2 in ARPE-19 cells, thereby further exploring the mechanics of the antioxidant effects of TP. As shown in Figure 4B, exposure to SI induced a significant decrease in p-PI3K/PI3K (*p* < 0.05), p-Akt/Akt (*p* < 0.05), and p-Nrf2/Nrf2 (*p* < 0.05), inhibiting the PI3K/Akt/Nrf2 signaling pathway. However, treatment of SI-exposed ARPE-19 cells with TP, notably increased the levels of p-Nrf2/Nrf2 (*p* < 0.05) (Figure 4E), p-Akt/Akt (*p* < 0.05) (Figure 4D), and p-PI3K/PI3K (*p* < 0.05) (Figure 4C). Two important intrinsic antioxidant molecules are HO-1 and NQO1. The protein content analysis in Figure 4B shows that the levels of HO-1 (*p* < 0.05) (Figure 4F) and NQO1 (*p* < 0.05) (Figure 4G) were lower in the SI group. Compared with the SI group, their levels in the TP group showed significantly elevated expressions (*p* < 0.01). The PI3K/Akt/Nrf2 pathway can be activated to increase the levels of HO-1 and NQO1 to reduce the damage caused by oxidative stress in ARPE-19 cells.

### TP-induced phosphorylation of Nrf2 protein

**Figure 5:**
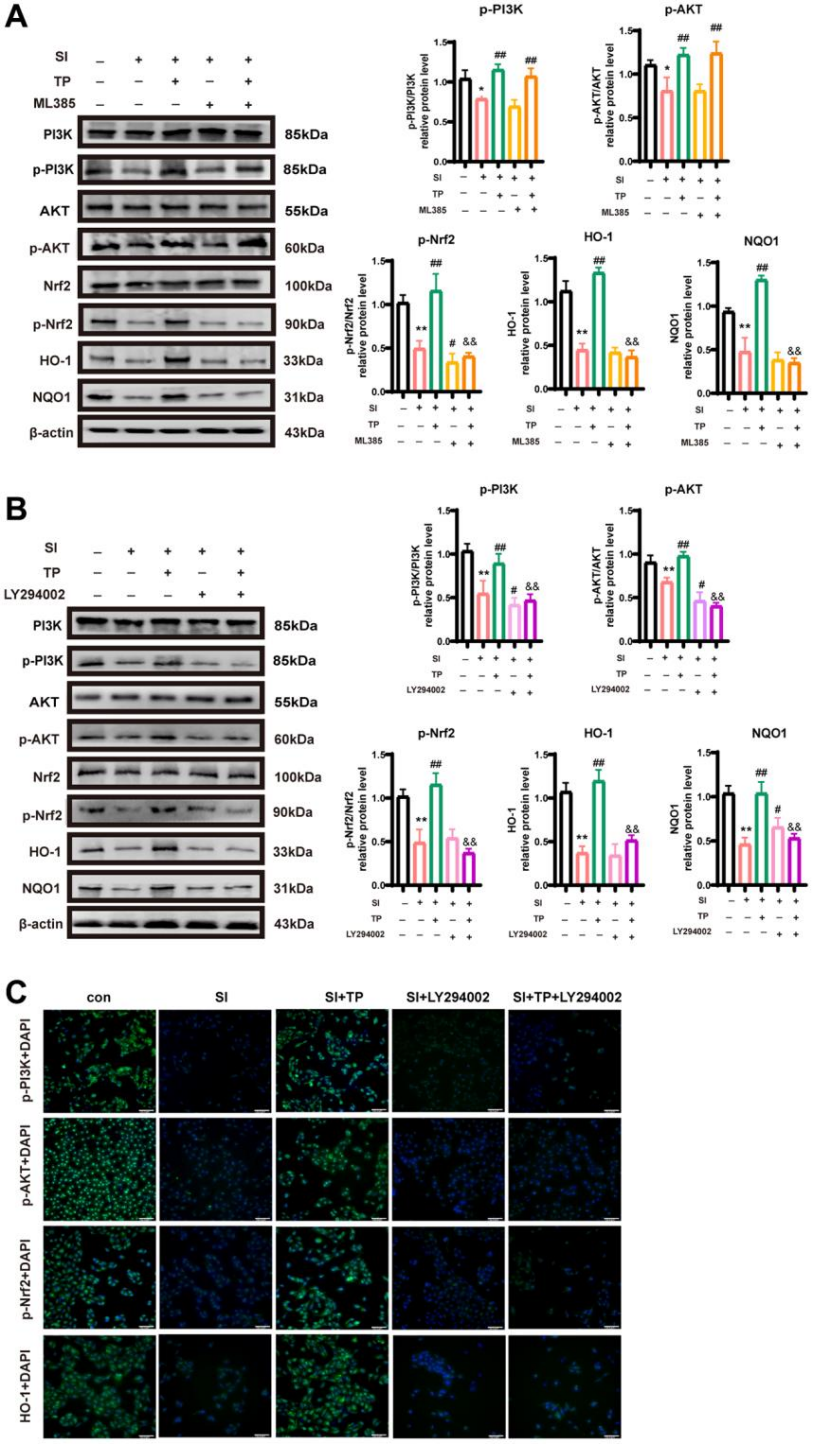
**Triptolide-activated PI3K/Akt/Nrf2 signaling pathway is helpful to the activation of antioxidant stress in SI-treated RPE cells.** Expression levels of p-PI3K/PI3K p-Akt/Akt, p-Nrf2/Nrf2, HO-1 and NQO1 relative proteins in ARPE-19 cells. The cells were incubated with LY294002 (10 μM) (**A**) or ML385 (1 μM) (**B**) for 1 h after pretreatment with TP (20 nM) for 6 h, then exposed to SI (10 mM) for 24 h. Quantitative analyses by ImageJ software (n = 3). (**C**) Immunofluorescence staining of p-PI3K, p-Akt, p-Nrf2 and HO-1 in ARPE-19 cells. Bar = 200 μm. Data are shown as mean ± standard deviation (SD) (*n* = 3); ^*^p < 0.05, ^**^P < 0.01 compared with the control group; ^#^*P* < 0.05, ^##^*P* < 0.01 compared with the SI group; ^&&^*P* < 0.01 compared with the SI+TP group.

Phosphorylation of Nrf2 protein induced by TP is regulated by the PI3K/Akt pathway. To confirm that Nrf2 protects ARPE-19 cells from SI-induced oxidative damage, we pretreated the cells with ML385 (1 μ mol/L), an Nrf2 inhibitor, and subsequently treated them with SI and/or TP. Compared with the treatment group, ML385 markedly reduced the levels of phosphorylated Nrf2 (p-Nrf2/β-actin) (*p* < 0.01), as evidenced by western blot analysis (Figure 5A). Meanwhile, the expression levels of HO-1 (*p* < 0.01) and NQO1 (*p* < 0.01) were reduced by ML385 (Figure 5A). In contrast, phosphorylation of Akt and PI3K was not influenced in Nrf2-inhibited cells compared with controls. (Figure 5A).

Additionally, LY294002 (10 μ mol/L) diminished the protective impact of the PI3K/Akt/Nrf2 pathway on SI-exposed ARPE-19 cells, as well as its activation by TP. The results in Figure 5B demonstrate that LY294002 suppressed the levels of p-Nrf2/Nrf2 (*p* < 0.01) and p-Akt/Akt (*p* < 0.01). LY294002 significantly inhibited PI3K/Akt phosphorylation and Nrf2 activation. Thus, these findings suggested that activation of the PI3K/Akt signaling pathway may be mediated through Nrf2 activation. As shown in Figure 5B, LY294002 significantly attenuated the protective effect of TP. Therefore, TP may protect ARPE-19 from oxidative stress by activating the PI3K/Akt/Nrf2 pathway. Furthermore, immunofluorescence assays showed that the levels of phosphorylated PI3K, phosphorylated Akt, phosphorylated Nrf2, and HO-1 dramatically increased with triptolide administration (Figure 5C). In addition, the triptolide-induced effects on the expression of p-PI3K, p-Akt, p-Nrf2 and HO-1 could be reversed by LY294002 (Figure 5C). The results of immunofluorescence (Figure 5C) were consistent with the in-cell western analysis. Flow cytometric analysis demonstrated that pretreatment with TP significantly increased the proportion of SI-induced the level of ROS, and this effect was eliminated by LY294002 treatment (*p* < 0.05) (Supplementary Figure 1B). These results indicate that PI3K/Akt activation is necessary for Nrf2 activation and the cytoprotective effects induced by TP. In conclusion, these results suggest that TP inhibits oxidative damage through the PI3K/Akt/Nrf2 signaling cascade.

## DISCUSSION

Age-related macular degeneration (AMD) is a chronic ocular condition associated with advancing age, which culminates in a significant impairment of visual function. Growing evidence suggests that cumulative oxidation accelerates the pathological process of AMD [30]. According to the two-stage model of AMD, oxidative stress is well recognized as the initial stage of molecular damage, which can then lead to a secondary stage characterized by oxidative explosion and inflammation [31]. Therefore, it is a feasible research direction to determine the therapeutic plant extracts for diseases related to oxidative stress. Studies in the DOMS rat model has demonstrated that natural products extracted from thunderbolt vine reduce pain, muscle inflammation, and oxidative stress without affecting liver function [32]. Therefore, in the current research, we assessed the role of TP in SI-induced oxidative stress in RPE cells through network pharmacology, molecular docking, and *in vitro* experiments.

Numerous academic studies have demonstrated that TP has effective antioxidant, anti-inflammatory, and antiproliferative activities [33]. Human clinical trials have been initiated for TP and its derivatives (PG490-88 and F60008) [34–36]. Nevertheless, the therapeutic use of TP is constrained due to its significant toxicity and low water solubility. *In vivo* studies have shown that the LD50 for IV-injected TP in mice is 0.83 mg/kg [37]. In Alzheimer’s disease model, TP at a concentration of 10^−10^ mol/L can protect against oxidative stress and apoptosis [38]. Therefore, we first tested the safety of triptolide on ARPE-19 cells before investigating the effect of triptolide. The results showed that a concentration of triptolide below 40 nM was safe in ARPE-19 cells. We found that SI induced concentration-dependent cytotoxicity in differentiated ARPE-19 cells, and that triptolide treatment in AMD models significantly increased cell viability compared with the SI group.

In addition, the accumulation of reactive oxygen species (ROS) is the main cause of retinal damage during the progression of the disease. SOD is an enzymatic scavenger of ROS, which is able to combat ROS accumulation and reduce oxidative damage [39]. The ability to prevent oxidative damage has always been associated with the activity level of SOD [40]. This research found that ROS levels in SI-exposed ARPE-19 cells were reduced, whereas SOD and CAT levels were raised after treatment with TP, showing that TP can significantly reduce ROS levels in ARPE-19 cells. Peroxidation of lipids, proteins, and DNA occurs with continuous exposure to light and oxidative stress. Furthermore, malondialdehyde (MDA), the major product of lipid peroxidation in AMD, can modify endogenous molecules and generate new oxidation-specific epitopes [41]. However, we observed that TP treatment reduced the level of MDA secretion in SI-exposed ARPE-19 cells. Altogether, this study supports the idea that TP can significantly reduce ROS levels in ARPE-19.

Furthermore, by constructing a network mode, network pharmacology can analyze the association between targets, drugs, and diseases [42, 43]. To determine the related targets of TP against AMD, we used network pharmacology. According to KEGG analysis, TP may play a beneficial role through the PI3K/Akt signaling pathway. The PI3K/Akt signaling pathway is often implicated in the upregulation of HO-1 expression and the activation of Nrf2-dependent transcription in several cell populations upon exposure to oxidative stress and other stimuli [44–46]. Our docking results showed that TP has high affinity to PI3K and Nrf2, further indicating that these two molecules may be the targets of TP in AMD. After receptor tyrosine kinase (RTK) interactions with growth factors, PI3K is normally activated. Akt serves as the downstream effector of PI3K. Following PI3K activation, Akt is recruited to the plasma membrane and phosphorylated [47]. Early studies also confirmed that PI3K/Akt pathway plays an essential part in cell survival and protection against oxidative stress and can increase Nrf2 stability by inhibiting glycogen synthase kinase-3β (GSK-3β) when activated [48–50].

**Figure 6:**
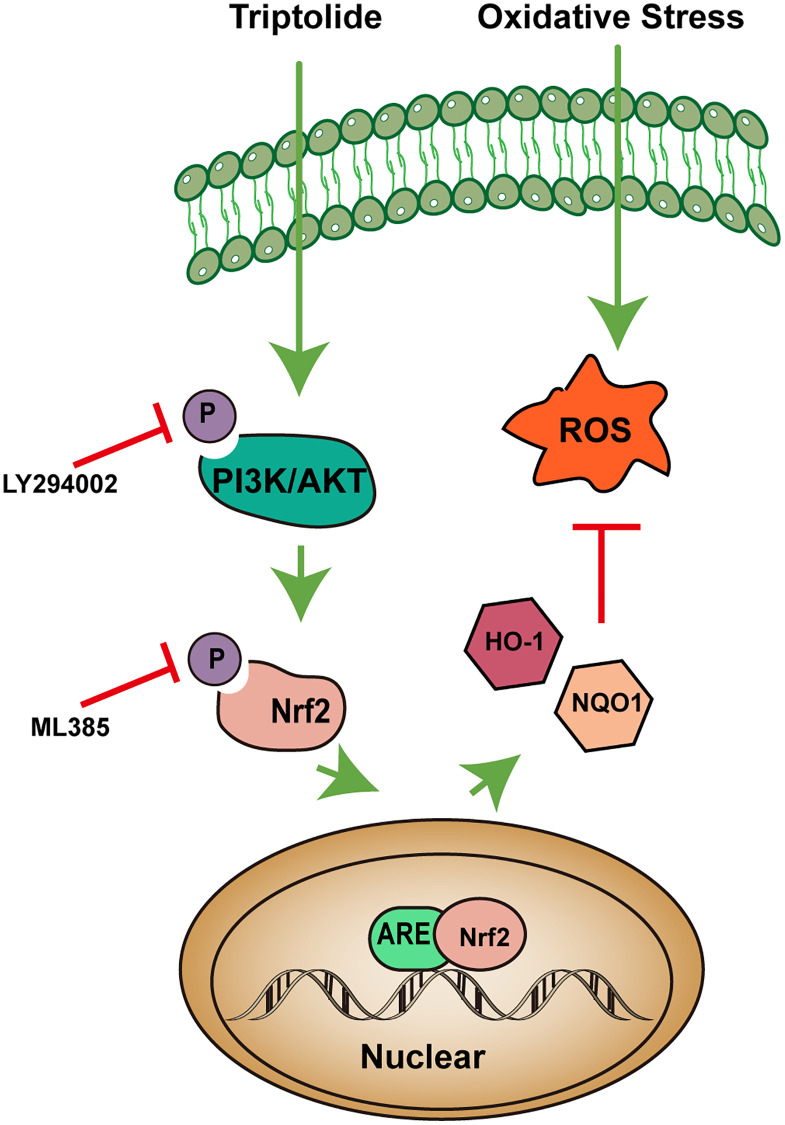
**A proposed signaling pathway involved in triptolide against oxidative stress (OS) in ARPE-19 cells.** The schematic diagram shows that TP induces Nrf2-mediated cytoprotective protein via activation of the PI3K/Akt signaling pathway, which protects against OS of ARPE-19 cells. Green arrows indicate stimulation, and red bars indicate inhibition.

Nrf2, a vital transcription factor for managing OS, can regulate antioxidant protein levels by interacting with antioxidant response elements (ARE) [51]. In this study, we found that the level of Nrf2 is lower in the serum of AMD patients compared with the control group. Under normal physiological conditions, Nrf2 binds to Keap1, a natural cytoplasmic inhibitor, to maintain activity and stability [52]. Nrf2 is activated and released from Keap1 following cellular injury and/or phosphorylation by various protein kinases. Research shows that the PI3K/Akt pathway is involved in phosphorylation of Nrf2 at serine 40. Inhibition of PI3K or Akt will attenuate the activation of Nrf2 [53]. It has also been proposed that another mechanism of Nrf2 dissociation from Keap1 involves the phosphorylation of serine/threonine residues in Nrf2 by various enzymes including casein kinase 2 (CK2), protein kinase C (PKC), and phosphoinositide 3-kinase (PI3K), which appear to control the nuclear import and export of Nrf2 [54, 55]. Phosphorylation promotes its translocation into the nucleus, followed by its heterodimerization with Maf; the heterodimer combines with ARE. Subsequently, transcription of a variety of antioxidant genes is upregulated in their promoter regions, including GSTs, HO-1, NQO1, and SODs [56, 57]. Through various enzyme-catalyzed reactions, these antioxidant molecules protect cells against oxidative stress. The findings of this study demonstrate that TP induces the activation of Nrf2 via the phosphorylation of PI3K/Akt protein kinase. Subsequently, this activation causes a rise in the expression of SOD, HO-1, NQO1, and other proteins, hence exerting inhibitory effects on oxidative stress-induced damage. Furthermore, inhibition of PI3K/Akt (via LY294002) reduced the levels of phosphorylated Nrf2, indicating that the antioxidant effect of TP depends on the activation of PI3K/Akt/Nrf2 signaling pathway. It has also been shown in the study that LY294002 reverses the expression of antioxidant molecules such as HO-1 [58]. In summary, by using a network pharmacology method and *in vitro* experiments, we were able to predict and verify that TP protects against oxidative stress in SI-exposed ARPE-19 cells. The antioxidant mechanism of TP may involve the stimulation of the PI3K/Akt signaling pathway, the facilitation of Nrf2 phosphorylation and its subsequent translocation into the nucleus, as well as the upregulation of HO-1 and NQO1 expression, as seen in Figure 6. The current study has several limitations and shortcomings. For example, SI-treated ARPE-19 cells are not an ideal model for all aspects of AMD. In addition, the establishment of an animal model of AMD is very important for the verification of the protective effect of TP, and these are the next research directions of our experimental group.

## CONCLUSION

In conclusion, our findings demonstrate that TP treatment effectively protects ARPE-19 cells from oxidative stress induced by SI by inhibiting ROS activity and activating the PI3K/Akt/Nrf2 signaling pathway. This information will enhance the understanding of TP in treating age-related macular degeneration and lays the foundation for future studies.

## MATERIALS AND METHODS

### Reagents and cell line

Triptolide was purchased from Topscience (Shanghai, China). Sigma Industrial Company (Shanghai, China) provided the sodium iodate, which was 98% pure. Anti-p-PI3K (Tyr458), anti-PI3K, anti-p-Akt (Ser473), anti-Akt, anti-Nrf2, anti-p-Nrf2 (Ser40), anti-HO-1, anti-NQO1, anti-Nanti-ZO-1, and anti-occludin were provided by Affinity Biosciences (Beijing, China). Cell Signaling Technology (Shanghai, China) was used to get anti-GAPDH and anti-actin. LY294002 (a PI3K/Akt inhibitor) and ML385 (an Nrf2 inhibitor) were obtained from Selleckchem (Houston, TX, USA). Human adult retinal pigment epithelium-19 (ARPE-19) cell line was acquired from the American Type Culture Collection (ATCC, Manassas, VA, USA). Dulbecco’s modified Eagle’s medium/nutrient mixture F12 (DMEM/F12) and antibiotics (penicillin and streptomycin) were acquired from Gibco (CA, USA). The source of fetal bovine serum (FBS) was GeminiBio (West Sacramento, CA, USA).

### ARPE-19 cell culture and cell viability assay

At 37°C, cells were cultured in a DMEM/F-12 solution with 10% FBS and 0.5% penicillin/streptomycin (10,000 U/mL^−1^) and 5% CO_2_ to keep the atmosphere moist. The effect and mechanism of TP on the SI-exposed ARPE-19 cells were measured by treating the cells with triptolide for 6 hours, then sodium iodate was added for 24 hours. LY294002 (10 μM/L) and ML385 (1 μM/L) were added independently for 1 hour before triptolide treatment.

The Cell Counting Kit-8 (Topscience, Shanghai, China) was used to evaluate the vitality of the cells. First, ARPE-19 cells (1 × 10^4^ cells/mL) were seeded in a 96-well plate (100 μL per well) and treated with sodium iodate or triptolide for 24 hours. In the same manner, the control well was established using medium without samples, while the blank well contained medium without cells. Cellular medium (100 μL per well) and CCK-8 reagent were added to the blank well and incubated for 2 hours at 37°C. At a wavelength of 450 nm, absorbance was measured using a microplate reader (Multiskan FC, Thermo Fisher Scientific, Waltham, MA, USA).

### Detection of intracellular ROS

Following the aforementioned treatment, the cells were subsequently incubated in a medium containing dichlorodihydrofluorescein diacetate (DCFH-DA) (Beyotime Institute of Biotechnology, Shanghai, China) at a concentration of 2.5 μM for 30 minutes. Images were captured by fluorescence microscope (Zeiss, Jena, Germany) and flow cytometry (Thermo Fisher Scientific, USA). ImageJ (NIH, Bethesda, MD, USA) was used to measure the ROS fluorescence intensity.

### Analysis of activities of SOD, CAT, and MDA

Following the aforementioned treatment, the cell supernatant was gathered and the overall activities of superoxide dismutase (SOD), catalase (CAT), and malondialdehyde (MDA) in ARPE-19 cells were analyzed using the respective kits (Beyotime Institute of Biotechnology, Shanghai, China). The experimental procedures followed the manufacturer’s specifications.

### EdU cell proliferation assay

Following the manufacturer’s recommendations, the EdU cell proliferation test was used to assess cell proliferation. Following treatment as above, the cells were labeled by adding 10 M of the EdU reagent (Beyotime, Shanghai, China) to each well, which was then incubated for 2 hours. After being washed with PBS, the cells were fixed in 4% paraformaldehyde solution (PFA, Beyotime, China) for 15 minutes, and then were permeabilized with 0.3% Triton X-100 (Sigma, USA) for another 15 minutes. Afterward, cells were incubated with click reaction mixture for 30 minutes at room temperature in the dark. To counterstain the nucleus, 1× Hoechst 33342 reagent was used. Confocal microscopy (Zeiss, Jena, Germany) images were taken.

### Measurement of mitochondrial membrane potential

We used JC-1 to perform the mitochondrial membrane potential (MMP) assay. In healthy cells with high mitochondrial membrane potential, a high level of JC-1 in the mitochondrial matrix causes red fluorescence. On the other hand, apoptotic cells have a lower MMP that facilitates the translocation of JC-1 into the cytosol, with its green fluorescence. Therefore, changes in JC-1 fluorescence can be used to detect changes in MMP. ARPE-19 cells were treated as above, washed with PBS, and then incubated in a medium containing JC-1 chromosomal solution at 37°C for 30 minutes. After two rinses with PBS, the change in MMP was detected using a confocal fluorescence microscope.

### Network pharmacology analysis

Potential targets of TP have been collected from Pharmmapper, TCMSP, Swiss TargetPrediction, and CTD databases and according to the results of Figure 1A. To find AMD-related targets, “age-related macular degeneration” was used as a keyword in GeneCards and OMIM databases. The online Venn diagram tool was then used to analyze TP-related targets and AMD-related targets for overlap. Protein-protein interaction (PPI) networks were constructed utilizing a string database, then were visualized using Cytoscape. Using the Metascape database and bioinformatics, GO and KEGG pathway enrichment analyses of key targets were performed.

### Molecular docking

Molecular docking using Autodock was performed to evaluate the interaction between TP and its targets. In a nutshell, the 3D structures of the TP targets were downloaded from the PDB protein database, and its 2D chemical structure was gathered from the PubChem database and transformed into 3D format with OpenBabel. After that, AutoDock software was used to simulate molecular docking, and PyMOL software was used to visualize the docking results.

### Determination of serum Nrf2

The study included 21 patients with AMD and 21 healthy controls. The diagnosis of AMD disease was confirmed in all patients based on well-defined diagnostic criteria. Our study excluded patients with diabetes mellitus, hypertension, dyslipidemia, a history of other autoimmune diseases, immune deficiencies, and other diseases. After explaining the nature and possible consequences of the study, informed consent was obtained from the subjects. An enzyme-linked immunosorbent assay (ELISA) kit (Proteintech, Wuhan, China) was used to measure serum Nrf2. The manufacturer’s protocol was followed for the assay.

### Western blotting

Protein was extracted with RIPA (Beyotime, Shanghai, China) using proteinase and phosphatase inhibitors (Roche, Basel, Switzerland), and then was centrifuged at 4°C for 10 minutes at 14,000 rpm. BCA protein assay (Beyotime, China) was employed to measure the concentration of the proteins. SDS-PAGE (Beyotime, China) was used to separate the protein samples, which were deposited afterward onto a PVDF membrane (Millipore, Burlington, MA, USA). At room temperature, the samples were then immersed in 5% skim milk for 1.5–2 hours. The primary antibody was then incubated on the membrane at 4°C for the following day. After three washes with TBST, the membrane was incubated with secondary antibody at room temperature for 2 hours. An ECL kit (Thermo Fisher Scientific, USA) was used for visualization, and signals were quantified using ImageJ software.

### Immunofluorescence analysis

ARPE-19 cells were seeded and then incubated in 24-well plates with glass coverslips overnight. Cells were incubated with sodium iodate and LY294002, fixed with chilled 4% paraformaldehyde for 15 minutes, permeabilized with 1% Triton for 20 minutes, and blocked with 10% goat serum for 30 minutes. Coverslips were then incubated overnight at 4°C with antibodies against HO-1 (1:200), p-Nrf2 (1:200), p-Akt (1:200), and p-PI3K (1:200). Then, goat anti-rabbit IgG conjugated with Alexa Fluor 488 (Beyotime, China) was incubated for 1 hour. DAPI staining was performed for 5 minutes. A confocal fluorescence microscope (Zeiss, Germany) was used for fluorescence imaging.

### Statistical analysis

Similar results have been obtained with 3 independent experiments. The program of IBM SPSS Statistics 20.0 (IBM, Armonk, NY, USA) was used to analyze the data, which were presented as mean ± SD. The one-way analysis of variance (ANOVA) was employed to analyze data from various groups. Additionally, unpaired Student’s *t*-test was performed to determine the significance between two groups. GraphPad Prism 9.0 software was used for all graphs and plots and *p* < 0.05 was considered significant.

## Supplementary Materials

Supplementary Figure 1
