# Supplementary material for: Protective effects of triptolide against oxidative stress in retinal pigment epithelium cells via the PI3K/AKT/Nrf2 pathway: a network pharmacological method and experimental validation
Source: Aging (Albany NY). 2024 Feb 21;16(4):3955–72. doi: 10.18632/aging.205570 (PMC10929812; doi:10.18632/aging.205570)
Supplement: Supplementary Figure 1 [file aging-16-205570-s001.pdf]

SUPPLEMENTARY FIGURE

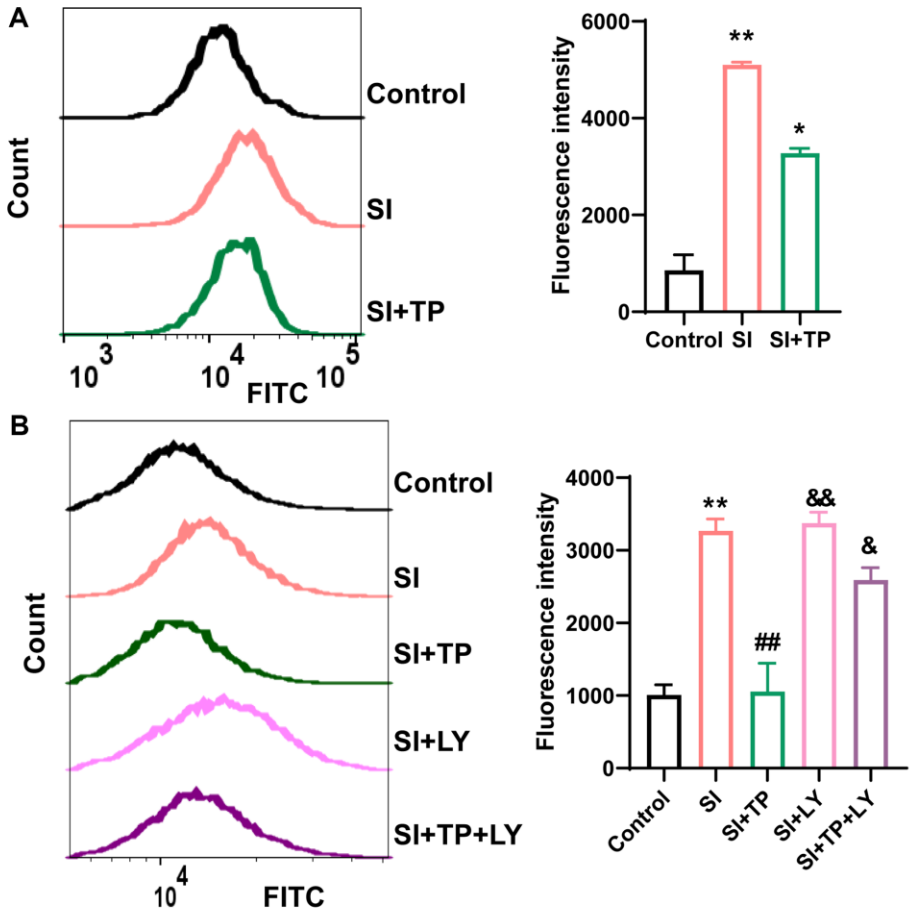

Supplementary Figure 1. TP had a rescue effect on the increased ROS caused by SI, and LY294002 (LY) reversed this rescue effect. After pretreatment with TP (20 nM) for 6 h (**A**), the cells were incubated with LY294002 (10 μM) for 1 h or not (**B**), then exposed to SI (10 mM) for 24 h. And the cells were stained with DCFH-DA to detect the intracellular ROS production by flow cytometry. Quantitative analyses by ImageJ software (n = 3). Data are shown as mean ± standard deviation (SD) (n = 3); $^{*}p < 0.05$, $^{**}P < 0.01$ compared with the control group; $^{\#\#}P < 0.01$ compared with the SI group; $^{\&}P < 0.05$, $^{\&\&}P < 0.01$ compared with the SI+TP group.
